# Pyrolysis temperature governs heavy metal immobilization by sewage sludge biochar in tropical multi-contaminated Vertisol: a multivariate assessment

**DOI:** 10.1007/s10653-026-03248-y

**Published:** 2026-05-15

**Authors:** Jorge Antonio Gonzaga Santos, Leiliane Oliveira dos Santos, Maria da Conceição de Almeida, Claudineia de Souza Souza, Oldair Del Arco Vinhas Costa, Thomas Vincent Gloaguen, Camila de Sena Magalhães, Fabiana Barbosa de Andrade, Henrique da Silva do Nascimento, Marcela Rebouças Bomfim

**Affiliations:** 1 Universidade Federal do Recôncavo da Bahia, Cruz das Almas, BA 44380-000 Brazil; 2 Universidade Federal da Bahia, Salvador, BA Brazil

**Keywords:** Biochar, Ecological risk assessment, Metal speciation, Principal component analysis, Soil remediation, Vertisol

## Abstract

This study evaluated the effectiveness of sewage sludge biochar (SSB) produced at 350, 450, and 600 °C for immobilizing potentially toxic elements (PTEs) in a tropical multi-contaminated Vertisol. Chromium, copper, cobalt, nickel, cadmium, lead, and zinc were assessed through sequential extraction, ecological risk indices, and principal component analysis (PCA). Pyrolysis temperature markedly altered biochar properties and, consequently, remediation performance. Among the treatments, SSB450 provided the most balanced and consistent response. It reduced the potential ecological risk index (RI) from 139.06 in the untreated soil to 65.23, representing an approximately 53.1% decrease, and produced the lowest RAC, Cf, PLI, and mCd values among the amended treatments. Increasing pyrolysis temperature promoted the redistribution of metals from mobile fractions (F1 + F2) to more stable fractions (F3 + F4). For example, the mobile fraction of Pb declined from 69.5% in the control soil to 35.2% after application of SSB450 and SSB600, while the mobile fraction of Cd decreased from 52.1% to 28.4% with SSB450. PCA confirmed clear multivariate separation among treatments and identified fixed carbon and organic carbon as the biochar attributes most strongly associated with lower contamination risk, with PC1 loadings of -0.916 and − 0.922, respectively. In contrast, SSB350 showed comparatively limited Cd stabilization, indicating that low-temperature biochar may be less suitable for uniform remediation of multi-contaminated Vertisols. Overall, the results demonstrate that careful calibration of pyrolysis temperature is essential to optimize the performance of sewage sludge biochar for heavy metal immobilization in tropical soils.

## Introduction

Soil contamination by potentially toxic elements (PTEs) is a persistent global environmental problem with direct implications for ecosystem functioning, food security, and human health. Unlike many organic pollutants, trace metals do not degrade and may remain in soils for decades or even centuries, thereby sustaining long-term ecological and toxicological risks (Bolan et al., 2014; Järup, 2003). In tropical regions, these risks are frequently intensified by rapid urbanization, mining, metallurgical residues, and historically inadequate waste-management practices.

Among the available remediation strategies, in situ immobilization has received increasing attention because it can reduce contaminant mobility and bioavailability without the high economic and environmental costs associated with soil excavation or off-site disposal (Kumpiene et al., 2008; Park et al., 2011). Biochar is particularly attractive in this context because its remediation potential derives from a combination of high surface area, alkaline reactivity, mineral ash, porous structure, and surface functional groups capable of promoting adsorption, ion exchange, complexation, and precipitation reactions (Ahmad et al., 2014; Inyang et al., 2016). In addition to contaminant stabilization, biochar may improve several soil attributes, including water retention, cation exchange capacity, and structural stability.

Sewage sludge is both an environmental liability and a potentially valuable feedstock for biochar production. Its conversion through pyrolysis reduces volume, sanitizes the material, and enables the recycling of carbon and mineral nutrients in a more stable form (Bridle & Pritchard, 2004; Fytili & Zabaniotou, 2008). However, the performance of sewage sludge biochar depends strongly on pyrolysis temperature, which controls pH, ash content, aromaticity, carbon stability, and the abundance of reactive surface groups (Cantrell et al., 2012; Hossain et al., 2011; Souza et al., 2021). Lower temperatures typically preserve more oxygen-containing functionalities but may yield less stable materials(Huang et al., 2017), whereas higher temperatures favor mineral concentration and structural ordering but can reduce the abundance of functional groups involved in specific metal-binding reactions (Beusch, 2021; Yameen et al., 2024).

This temperature dependence is especially relevant in Vertisols, which are clay-rich soils characterized by shrink-swell behavior, high cation exchange capacity, and complex redox and drainage dynamics. Despite their agricultural importance in tropical and subtropical regions, Vertisols remain underrepresented in the biochar remediation literature. The present study addresses this gap by evaluating sewage sludge biochars produced at 350, 450, and 600 °C for the immobilization of Cr, Cu, Co, Ni, Cd, Pb, and Zn in a tropical multi-contaminated Vertisol from Bahia, Brazil. Specifically, the study aimed to: (i) determine how pyrolysis temperature modifies biochar properties; (ii) assess changes in metal speciation using sequential extraction; (iii) quantify ecological risk using complementary contamination indices; and (iv) identify the treatment with the best overall performance through multivariate analysis.

## Materials and methods

### Site description and soil sampling

Soil samples were collected from a multi-contaminated site in the Recôncavo region of Bahia State, Brazil (12°40'S, 39°06'W), in an area with a documented history of anthropogenic contamination associated with industrial activities and metallurgical residues. The region has a tropical climate (Köppen Af), with a mean annual temperature of 24.5 °C and annual rainfall of approximately 1200 mm. According to Soil Taxonomy, the soil is classified as a Vertisol (USDA, 2022).

Composite samples were collected from the 0–20 cm layer at five randomly selected points, thoroughly homogenized, air-dried, and passed through a 2-mm sieve. Preliminary screening indicated elevated concentrations of Cd, Pb, Cu, Zn, Cr, Ni, and Co relative to Brazilian soil quality guidelines, confirming the multi-element contamination status of the site (Brasil, 2009).

### Sewage sludge collection and biochar production

Anaerobically digested sewage sludge (SS) was obtained from a municipal wastewater treatment plant in Cruz das Almas, Bahia, Brazil. After air-drying, the material was ground to pass a 1-mm sieve and subjected to slow pyrolysis in a laboratory-scale fixed-bed reactor under a continuous N2 flow of 150 mL min^−1^. Biochars were produced at 350 °C (SSB350), 450 °C (SSB450), and 600 °C (SSB600), using a heating rate of 10 °C min-1 and a residence time of 2 h at the target temperature. The resulting biochars were cooled under N2, ground, sieved to < 0.5 mm, and stored in airtight containers until use.

### Biochar characterization

Biochar pH and electrical conductivity (EC) were determined in deionized water using a 1:10 (m/v) suspension after 30 min of agitation and 5 min of settling. The supernatant’s pH and EC were measured using Orion Versa Star Pro 40 Multiparameter Meter for pH/ISE. Cation exchange capacity (CEC) was determined by NH4 + adsorption followed by displacement with KCl, as described by Aston et al. (2013), and NH4 + was quantified spectrophotometrically (PerkinElmer Lambda 25 UV/Vis) at 400 nm using the Nessler method. The CEC was calculated using the Eq. 1:

1 $$CEC=N{H}_{4(extractant)}^{+}+N{H}_{4(blank)}^{+}/14$$

Proximate analysis included moisture, volatile matter, ash, and fixed carbon. Moisture was determined after heating to 150 °C, volatile matter after heating from 150 to 950 °C, and ash after combustion at 750 °C for 6 h in a muffle furnace (Linn-Elektro Therm model N 480 D). Fixed carbon (Eq. 2) was estimated by difference. Elemental composition (C, H, N, and S) was measured using an elemental analyzer (Vario EL III, Elementar, Hanau, Germany), and O was calculated by difference (Eq. 3). Mineral composition was assessed by portable X-ray fluorescence spectrometry (PXRF; Bruker Titan 600 LE) according to USEPA Method 6200 (US EPA, 2007), using triplicate measurements and NIST-certified reference materials for quality control.

2 $$FC(\%)=100\%-(moisture(\%)-volatilematter(\%)-ash.(\%))$$

3 $$\%O=100-(\%C+\%H+\%N+\%S+\%ash)$$

### Experimental design and soil treatment

The pot experiment followed a completely randomized design with four treatments and five replicates per treatment: untreated contaminated soil (control), soil + SSB350, soil + SSB450, and soil + SSB600. Each experimental unit contained 300 g of air-dried Vertisol. Before amendment addition, the soil was adjusted to 60% of field capacity and pre-incubated for 30 days at 25°C in a biochemical oxygen demand incubator (SOLAB SL-224). Each treatment comprised five replicates. Pot positions were rotated weekly to minimize environmental gradients within the chamber.

Biochar was applied at 5% (w/w), after passage through a 2-mm sieve, and thoroughly mixed with the pre-incubated soil. The amended soils were then incubated for 365 days at approximately 60% field capacity, which was maintained by periodic weighing and compensation with deionized water. The extended incubation period was selected because biochar produced at higher pyrolysis temperatures (> 500 °C) typically exhibits a highly aromatic and stable carbon structure, requiring longer aging to develop surface functional groups and charge. Pots were kept partially open to permit aeration while minimizing excessive evaporation and leaching. Redox dynamics and leachate losses were not directly monitored during incubation. At the end of the incubation period, the samples were air-dried and used for physicochemical, fractionation, and ecological risk analyses.

### Soil physicochemical characterization

Baseline soil chemical and physical analyses were performed according to standard procedures described by Teixeira et al., (2017). Particle-size distribution was determined by the pipette method. Soil pH was measured in water and 1 mol L^−1^ KCl using a 1:2.5 soil-to-solution ratio. Exchangeable Ca^2+^, Mg^2+^, and Al^3+^ were extracted with 1 mol L^−1^ KCl, whereas potential acidity (H + Al^3+^) was determined after extraction with 0.5 mol L^−1^ calcium acetate at pH 7.0. Sodium and potassium were measured by flame photometry (Digimed DM-62), and available P was determined using Mehlich-1 extraction followed by colorimetry (Cary, Varian). Sum of bases, CEC, and base saturation were calculated from these data. Soil organic carbon was measured by dichromate oxidation (Teixeira et al., 2017). The contaminated Vertisol showed the following baseline properties: clay content, 68.2 ± 3.4%; pH(H2O), 6.8 ± 0.2; pH(KCl), 5.9 ± 0.3; organic carbon, 12.4 ± 1.1 g kg^−1^; CEC, 28.7 ± 2.3 cmolc kg^−1^; and available P, 8.2 ± 0.9 mg kg^−1^.

### Sequential extraction and ecological risk assessment

Metal fractionation in the treated soils was determined using the BCR sequential extraction scheme (Ure et al., 1993), which operationally separates metals into the acid-soluble/exchangeable fraction (F1), reducible fraction (F2), oxidizable fraction (F3), and residual fraction (F4). The residual fraction was obtained after digestion according to USEPA Method 3050B (USEPA, 1996). Because the BCR procedure yields operationally defined fractions, treatment effects were interpreted comparatively rather than as direct evidence of discrete mineral phases.

Metal concentrations in each fraction were measured by ICP-OES (Agilent 720). Quality assurance included analysis of certified reference material BCR-701, with recoveries between 92 and 108%. Each biological replicate was analyzed with three analytical replicates.

Ecological risk and contamination indices were calculated from the sequential extraction data using the following Eqs. 4–10. Because these indices were derived from operationally defined fractionation data, they are interpreted here as comparative indicators of relative mobility-associated risk rather than as direct equivalents of classical background-based ecological risk metrics.

4 $${\text{Cf }} = \frac{F1 + F2 + F3}{{F4}}$$

5 $$ER=Tr x Cf$$

6 $$RI= \sum ER$$

7 $$RAC=\left(\frac{F1}{\sum \left(F1+F2+F3+F4\right)}\right)x 100$$

8 $$Cd= \sum Cf$$

9 $$mCd= \sum Cf/n$$

10 $$PLI= \sqrt[n]{CF1XCF2XCF3\dots .CFn}$$

In these equations, Cf is the contamination factor for each metal (Angelova et al., 2025), ER is the individual ecological risk factor, RI is the integrated potential ecological risk index, RAC is the risk assessment code, Cd is the contamination degree, mCd is the modified contamination degree, and PLI is the pollution load index. Toxic-response factors (Tr) were Zn = 1, Cr = 2, Cu = 5, Co = 5, Ni = 5, Pb = 5, and Cd = 30, following the risk assessment framework used in the original study. Index interpretation followed the classification system described in the source manuscript.

Cf values were classified as < 1 (low), 1–3 (moderate), 3–6 (considerable), and > 6 (very high). Toxic-response factors were Zn = 1, Cr = 2, Cu = Co = Ni = Pb = 5, and Cd = 30. RI values were interpreted as < 150 (low risk), 150–300 (moderate risk), 300–600 (considerable risk), and > 600 (very high risk). Five types of RAC risks were defined no risk (NR, RAC < 1%), low risk (LR, 1% ≤ RAC < 10%), medium risk (MR, 10% ≤ RAC < 30%), high risk (HR, 30% ≤ RAC < 50%), and very high risk (VHR, RAC ≥ 50%); Cd is the sum of CF; and mCd), where ‘n’ is the number of metals analyzed; and PLI < 1 (no pollution), and PLI > 1 (pollution).

### Statistical analysis

Data normality and homoscedasticity were checked using the Shapiro–Wilk and Levene tests, respectively. Treatment means were compared by one-way ANOVA followed by Tukey's HSD test at *p* < 0.05.

Principal component analysis (PCA) was used to synthesize multivariate relationships among treatments, ecological risk indices, and biochar properties. PCA was performed using (treatment means / individual replicate data) after z-score standardization of the selected variables. Components were retained according to eigenvalues > 1, scree-plot inspection, and cumulative explained variance. To improve interpretability, Varimax rotation was applied to the retained components, and variables with |loading|≥ 0.75 were considered influential.

## Results

### Biochar physicochemical properties

Pyrolysis temperature markedly changed the physicochemical characteristics of the sewage sludge-derived biochars, Tables 1, 2. Relative to untreated sewage sludge, all biochars showed higher pH, EC, CEC, ash content, and fixed carbon, whereas moisture and volatile matter decreased progressively with increasing temperature. The pH rose from 6.8 in SS to 8.2, 9.1, and 10.4 in SSB350, SSB450, and SSB600, respectively. Likewise, CEC increased from 18.4 to 22.7, 28.9, and 31.6 cmolc kg^−1^, indicating a temperature-driven enhancement in reactive surface properties.

**Table 1:** Physicochemical characteristics and proximate analysis of sewage sludge and derived biochars

Property	SS	SSB350	SSB450	SSB600
pH	6.8 ± 0.1	8.2 ± 0.2	9.1 ± 0.2	10.4 ± 0.3
EC (mS cm-1)	2.1 ± 0.3	3.4 ± 0.4	4.7 ± 0.5	6.8 ± 0.6
CEC (cmolc kg-1)	18.4 ± 2.1	22.7 ± 2.8	28.9 ± 3.2	31.6 ± 3.5
Moisture (%)	8.2 ± 0.4	5.1 ± 0.3	3.2 ± 0.2	2.1 ± 0.1
Volatile matter (%)	68.5 ± 2.1	45.3 ± 1.8	32.7 ± 1.5	24.1 ± 1.2
Fixed carbon (%)	7.42 ± 0.8	9.84 ± 1.1	11.60 ± 1.3	13.18 ± 1.5
Ash (%)	15.9 ± 1.2	39.5 ± 2.1	52.4 ± 2.8	60.6 ± 3.2

**Table 2:** Ultimate analysis of sewage sludge and derived biochars

Element	SS	SSB350	SSB450	SSB600
C (%)	25.15 ± 1.2	20.31 ± 1.0	18.51 ± 0.9	16.17 ± 0.8
H (%)	4.41 ± 0.3	1.42 ± 0.1	1.01 ± 0.1	0.73 ± 0.1
N (%)	4.44 ± 0.2	3.51 ± 0.2	3.06 ± 0.2	2.86 ± 0.1
S (%)	1.47 ± 0.1	1.33 ± 0.1	1.22 ± 0.1	1.16 ± 0.1
O* (%)	4.07 ± 0.4	2.41 ± 0.3	1.46 ± 0.2	0.71 ± 0.1
H/C ratio	2.104	0.840	0.656	0.542
O/C ratio	0.122	0.089	0.058	0.032

^*^Calculated by difference

Elemental analysis further showed progressive declines in C, H, N, S, and O contents and lower H/C and O/C ratios in the biochars, consistent with increasing thermal condensation and structural stabilization.

### Redistribution of metals among geochemical fractions

**Fig. 1:**
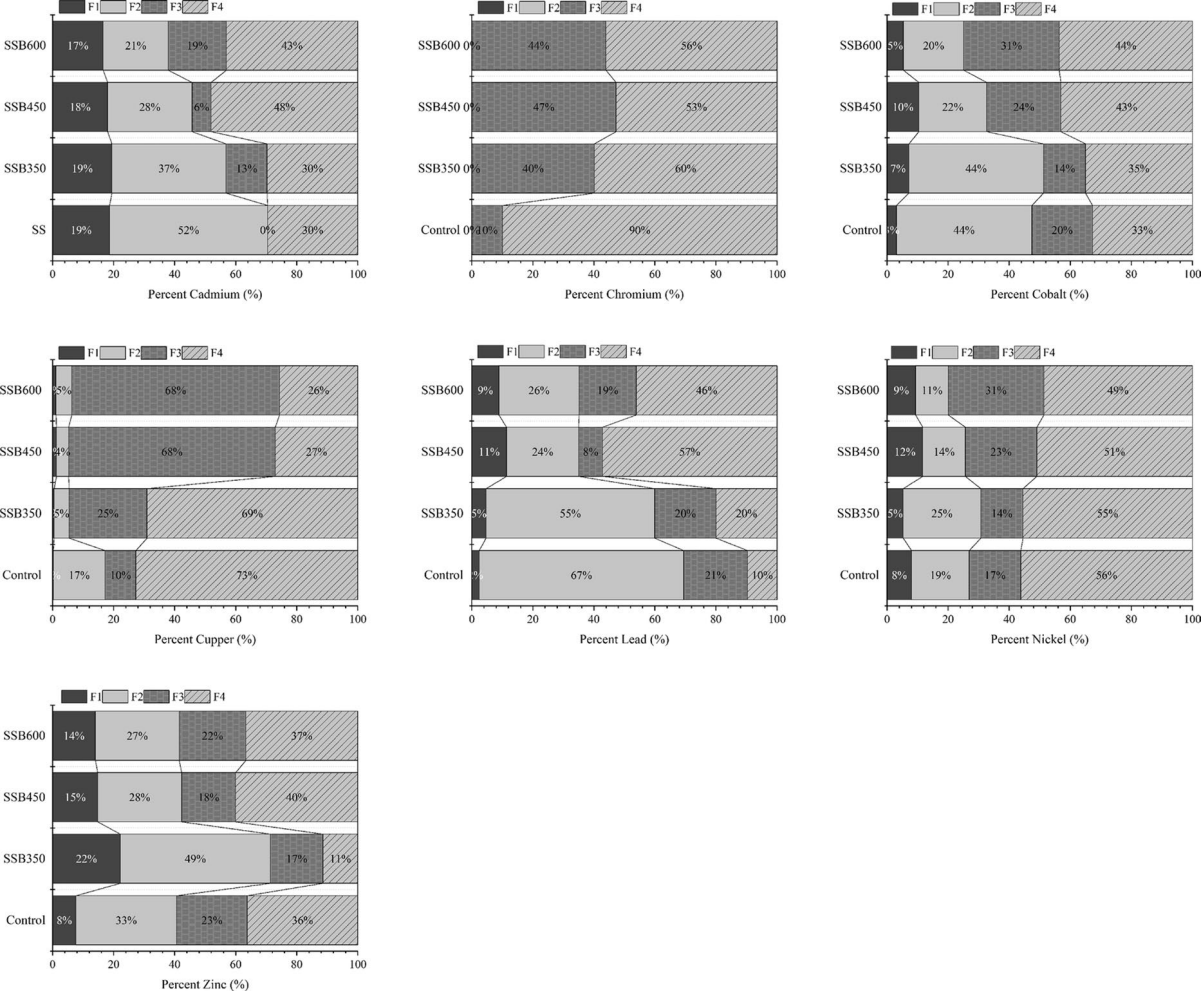
Distribution of heavy metal fractions in sewage sludge (SS) and sewage biochar pyrolyzed at of 350, 450, and 600°C temperatures (SSB_350_, SSB_450,_ and SSB_600_, respectively)

Biochar amendment promoted the redistribution of several metals from mobile fractions (F1 + F2) toward more stable fractions (F3 + F4), although the magnitude of this effect varied among elements and pyrolysis temperatures, Fig. 1**.** Lead showed one of the clearest responses: its mobile fraction decreased from 69.5% in the untreated soil to 60.0% with SSB350 and to 35.2% with both SSB450 and SSB600. Cadmium followed the same trend, decreasing from 52.1% in the control to 38.6%, 28.4%, and 31.2% under SSB350, SSB450, and SSB600, respectively. Copper and Zn also became less concentrated in mobile or acid-soluble pools after biochar application, whereas Co and Ni exhibited more moderate redistribution. Chromium remained predominantly associated with the residual fraction across all treatments, indicating limited mobility irrespective of amendment.

### Ecological risk indices

**Fig. 2:**
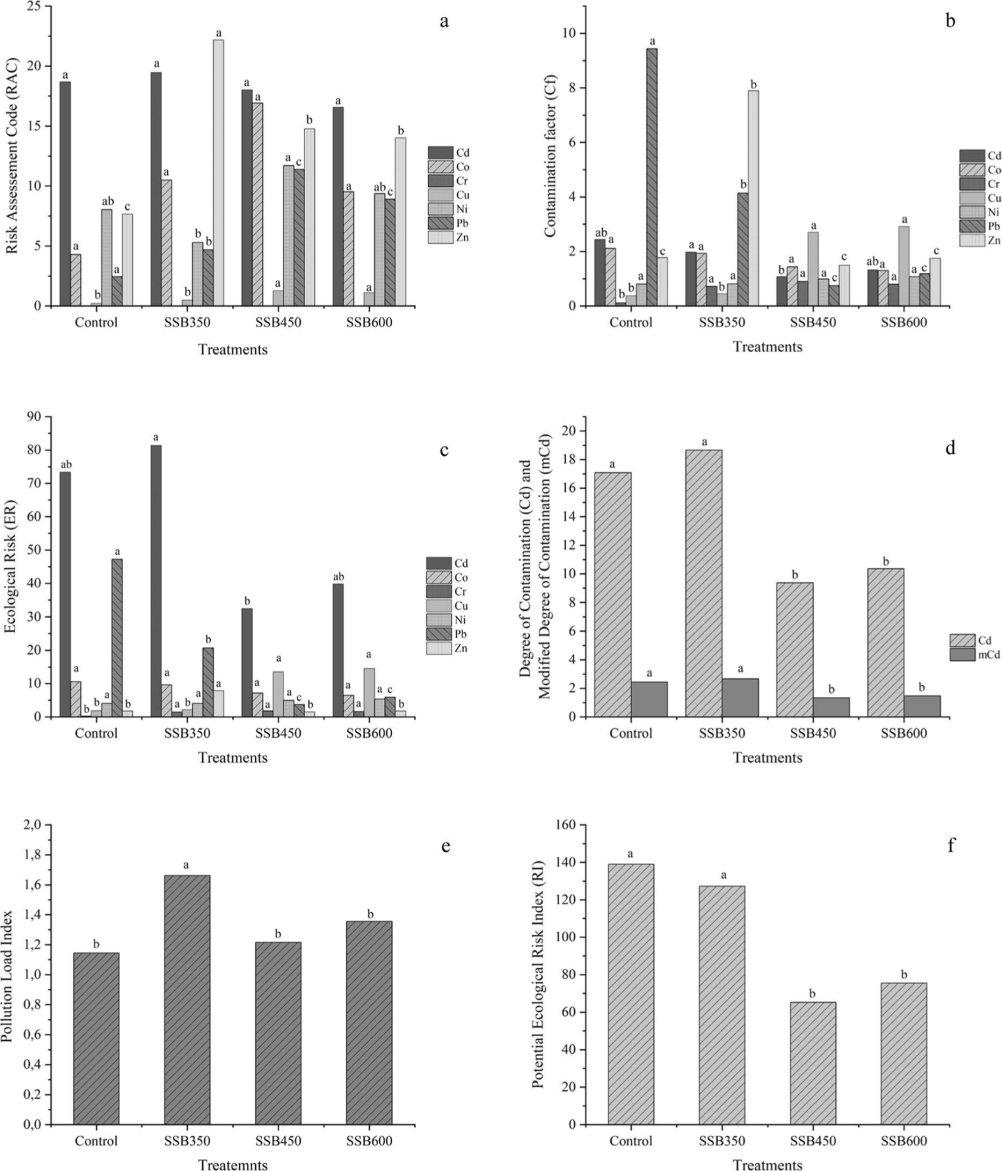
Influence of Sewage Sludge Biochar (SSB) pyrolyzed at 350 (SSB350), 450 (SSB450) and 600 (SSB600) in different ecological indicators: **a** Risk Assessment Code; **b** Contamination Factor; **c** Ecological Risk; **d** Cd and mCd; **e** Pollution Load Index; **f** Potential Ecological Risk Index (RI) in the Soil multi-contaminated (Control)

The shift in metal partitioning was accompanied by lower ecological risk across all biochar treatments. The strongest improvements were observed for Cd and Pb. For Cd, RAC declined from 28.3% in the control soil to 19.2% with SSB350, 8.7% with SSB450, and 11.3% with SSB600. For Pb, RAC decreased from 24.7% to 12.4%, 6.3%, and 7.8%, respectively, Fig. 2a. Contamination factors followed the same pattern: Cd Cf declined from 4.8 in the control to 3.6, 2.1, and 2.5, while Pb Cf decreased from 3.2 to 2.4, 1.3, and 1.6 (2b). At the integrated level, RI declined from 139.06 in the untreated soil to 105.28, 65.23, and 75.52 in SSB350, SSB450, and SSB600, respectively (Fig. 2c). PLI (Fig. 2e) and mCd (Fig. 2d) also decreased consistently after amendment, again identifying SSB450 as the most effective treatment.

### Principal component analysis

**Fig. 3:**
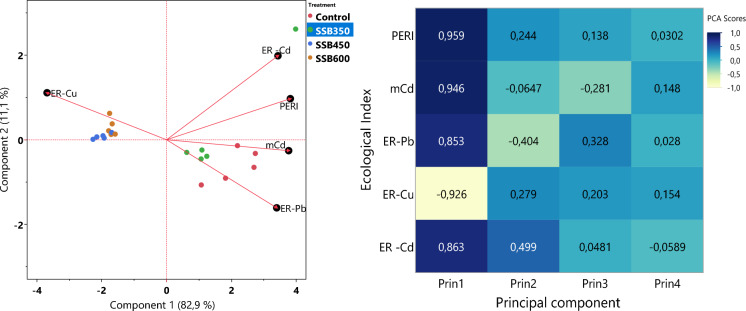
Biplot **a** and heatmap **b** of the Ecological Risk indicators with their respective loadings by components

PCA highlighted clear overall multivariate treatment dataset comparison. For the ecological risk dataset, the first two principal components explained 94.0% of the total variance, with PC1 and PC2 accounting for 82.9 and 11.1%, respectively, Fig. 3a. Within PC1, RI (PERI) and mCd showed the highest positive loadings (0.959 and 0.946, respectively), indicating that these variables contributed most strongly to the ecological risk gradient. PC1 scores were + 2.338 for the control, + 2.397 for SSB350, − 2.554 for SSB450, and − 1.652 for SSB600, indicating that SSB450 was associated with the lowest overall contamination-risk profile.

For the biochar-property PCA, the first two components explained 96.2% of the variance (PC1 = 89.5%; PC2 = 7.7%), Fig. 3a. Fixed carbon and organic carbon showed the strongest negative PC1 loadings (-0.916 and -0.900, respectively), indicating that these attributes were strongly associated with lower contamination and ecological risk.

## Discussion

### Pyrolysis temperature controlled the functional properties of sewage sludge biochar

The results demonstrate that pyrolysis temperature was the dominant factor regulating the properties of sewage sludge biochar and, consequently, its remediation performance. The increase in pH, EC, ash content, and CEC with increasing temperature is consistent with the concentration of mineral constituents and the progressive loss of volatile organic components during pyrolysis (Cantrell et al., 2012; Hossain et al., 2011; Yuan et al., 2011). Simultaneously, the decline in H/C and O/C ratios indicates greater aromatic condensation and structural stability at higher temperatures, which is typically associated with enhanced persistence in soil (Enders et al., 2012).

These changes are directly relevant for metal immobilization. Mineral-rich and increasingly alkaline biochars can promote precipitation and surface retention of cationic metals, whereas higher fixed-carbon content contributes to the development of persistent sorption domains while residual organic carbon supplies functional groups (carboxyl, hydroxyl, phenolic) for metal complexation (Karimi et al., 2020; Li et al., 2025). However, greater thermal severity is not invariably advantageous. Excessive temperature can reduce oxygen-containing functional groups that are important for specific surface complexation reactions. The present dataset therefore supports a balanced interpretation: both structural maturation and retention of reactive surface chemistry are important, and optimal remediation should be expected at an intermediate rather than an extreme pyrolysis temperature.

### Biochar amendment shifted metals toward less mobile forms

**Fig. 4:**
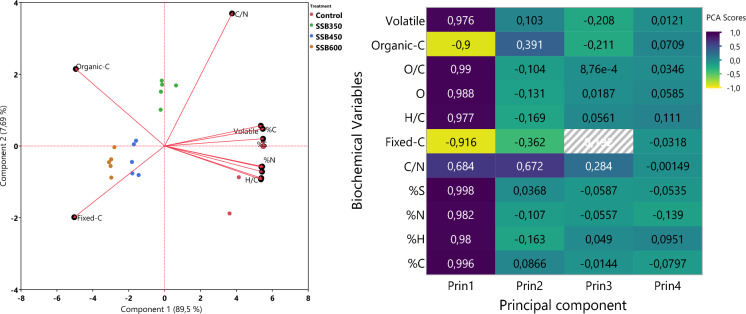
Biplot **a** and heatmap **b** of the biochemical variables with their respective loadings by components

The fractionation results show that sewage sludge biochar effectively reduced the mobility of several metals, especially Cd and Pb. The pronounced decline in the mobile Pb fraction after SSB450 and SSB600 suggests strong affinity between Pb and biochar-associated mineral and reactive surface phases. This behavior agrees with previous studies reporting that Pb is readily immobilized by Fig. 4 biochar through adsorption, precipitation, and association with phosphate- and carbonate-rich ash components (Houben et al., 2013; Karimi et al., 2020; Uchimiya et al., 2010).

Cadmium was more sensitive to pyrolysis temperature than Pb. Although all biochars reduced Cd mobility relative to the control, SSB450 provided the strongest stabilization, whereas SSB350 was notably less efficient. This pattern is important because Cd is among the most ecotoxic and mobile elements in contaminated soils. Similar temperature-dependent behavior has been reported for sludge-derived biochars, in which incomplete stabilization at lower pyrolysis temperatures may limit remediation effectiveness or even maintain relatively labile Cd pools (Li et al., 2024; Sørmo et al., 2024).

The weaker responses observed for Co and Ni and the largely residual behavior of Cr indicate that biochar effects were element-specific. Such differences are expected because metal partitioning depends not only on biochar properties but also on each element's intrinsic geochemical behavior, oxidation state, and affinity for mineral and organic phases. Accordingly, the remediation value of a given biochar should be judged on the basis of a multi-element perspective rather than a single-metal response.

### Plausible mechanisms of heavy metal immobilization

The observed redistribution patterns are consistent with the operation of multiple, complementary immobilization mechanisms. First, oxygen-containing functional groups and aromatic domains on the biochar surface likely promoted adsorption and complexation reactions. Recent mechanistic work has also suggested that cation-pi interactions may contribute to Cd retention on sludge-derived biochars (Li et al., 2025). Second, the alkaline and ash-rich nature of the biochars probably favored the precipitation or co-precipitation of metal hydroxides, carbonates, and phosphates, particularly for Pb and Cd (Ahmad et al., 2014; Zhang & Tsang, 2019).

Third, the increase in CEC indicates greater availability of exchange sites capable of temporarily retaining cationic metals, thereby lowering their activity in soil solution. Fourth, the more developed carbon structure at intermediate and higher temperatures may have enhanced physical entrapment within the pore network. Finally, shifts toward the reducible fraction suggest that biochar may also facilitate the association of metals with Fe and Mn oxide phases, either directly or indirectly through changes in soil chemistry (Wang et al., 2022). Rather than acting through a single pathway, sewage sludge biochar appears to immobilize metals through a combination of sorption, ion exchange, precipitation, and phase association processes.

### Why SSB450 showed the most balanced overall performance

The central finding of this study is that SSB450 outperformed both SSB350 and SSB600 when all response variables were considered together. This superiority was evident not only in the fractionation data but also in the integrated risk indices and PCA results. SSB450 yielded the lowest mobile Cd fraction, the lowest Cd and Pb RAC values, the lowest RI, PLI, and mCd, and the most negative PC1 score in the ecological-risk PCA. The convergence of these independent metrics strengthens the conclusion that 450 °C was the most suitable pyrolysis temperature among those tested.

Mechanistically, SSB450 appears to represent a compromise between insufficient thermal transformation and excessive thermal severity. Relative to SSB350, the 450 °C treatment likely achieved greater mineral concentration, alkalinity, and structural stabilization. Relative to SSB600, it likely retained a more favorable balance of reactive surface functionality while still providing substantial fixed carbon and ash-mediated retention capacity. In this sense, the results argue against a simple assumption that higher temperature always leads to better remediation performance. Instead, they indicate that optimal temperature is the one that maximizes the combined benefits of structural development, mineral reactivity, and chemically active surface sites.

### Environmental implications for tropical Vertisols

From an applied perspective, the results are promising for the remediation of contaminated tropical Vertisols. All biochar treatments reduced the integrated ecological risk to the low-risk class (RI < 150), indicating that sewage sludge biochar can be a practical amendment for in situ risk mitigation in this soil type. The effect is especially relevant for Cd and Pb, whose high toxicity and mobility make them priority contaminants in environmental risk management. Reduced mobility also implies lower susceptibility to plant uptake and leaching, both of which are critical concerns in agricultural landscapes.

The findings also support the broader concept of sludge valorization within a circular-economy framework. Converting sewage sludge into biochar offers a pathway to transform a difficult waste stream into a functional material for environmental remediation. Nevertheless, the present results also highlight that product quality cannot be assumed solely from the feedstock. Pyrolysis temperature decisively affected performance, and low-temperature biochar was less reliable for Cd stabilization. Therefore, remediation-oriented use of sludge-derived biochar should be coupled with strict production control and post-production characterization.

### Study limitations and future research

Although the study provides a robust comparison among pyrolysis temperatures, several limitations should be acknowledged. First, the experiment was conducted under controlled pot conditions using a single tropical Vertisol, so the findings should not be extrapolated uncritically to other soil classes or field settings. Second, the assessment focused on fractionation and ecological risk indices rather than direct measurements of leaching, crop uptake, or long-term weathering resistance. Third, mechanistic interpretations are supported by the observed patterns and the literature, but they were not directly validated by complementary spectroscopic or mineralogical analyses.

Future studies should therefore include long-term field trials, leaching assays, plant uptake assessments, and weathering experiments under tropical conditions. Additional work on surface area, functional-group chemistry, and mineral transformations would also help clarify the mechanisms responsible for the superior performance of SSB450. Finally, optimization of other pyrolysis parameters, such as residence time, heating rate, and feedstock pre-treatment, could refine the production of sludge-derived biochars tailored to specific contamination scenarios.

## Conclusions

Pyrolysis temperature was the key variable governing the remediation performance of sewage sludge biochar in the studied tropical Vertisol. All biochar treatments reduced metal mobility and ecological risk relative to the untreated soil, but SSB450 delivered the most consistent and balanced response across the evaluated indicators. This treatment reduced RI from 139.06 to 65.23, markedly lowered the RAC and contamination factors of Cd and Pb, and showed the clearest multivariate separation from the control and the lower-performing treatments. The results further indicate that increasing pyrolysis temperature promotes the transfer of metals from mobile fractions to more stable pools, but that the best remediation outcome is achieved at an intermediate temperature rather than at the highest temperature tested.

The PCA results identified fixed carbon and organic carbon as the biochar attributes most strongly associated with lower contamination risk, reinforcing the importance of both structural stability and reactive surface chemistry in metal immobilization. Overall, the study provides practical evidence that sewage sludge biochar can be used as a remediation-oriented amendment in tropical contaminated soils and that careful temperature calibration is essential to maximize its effectiveness and environmental safety.

## Supplementary information

We acknowledge that the Availability of Data and Materials section was missing in the original submission. This section has now been added to the Declarations and specifies how the data supporting the findings of this study can be accessed.

## Data Availability

No datasets were generated or analysed during the current study.
